# Systematic unravelling of the inulin hydrolase from *Bacillus amyloliquefaciens* for efficient conversion of inulin to poly-(γ-glutamic acid)

**DOI:** 10.1186/s13068-019-1485-9

**Published:** 2019-06-13

**Authors:** Yibin Qiu, Yifan Zhu, Yijing Zhan, Yatao Zhang, Yuanyuan Sha, Yijing Zhan, Zongqi Xu, Sha Li, Xiaohai Feng, Hong Xu

**Affiliations:** 1 State Key Laboratory of Materials-Oriented Chemical Engineering, Nanjing, 211816 China; 2 College of Food Science and Light Industry, Nanjing Tech University, Nanjing, 211816 China; 3 Nanjing Shineking Biotech Co., Ltd, Nanjing, 210061 China

**Keywords:** *Bacillus amyloliquefaciens*, Inulin hydrolase, Poly-(γ-glutamic acid), Jerusalem artichoke biorefinery

## Abstract

**Background:**

*Bacillus amyloliquefaciens* NB is a newly discovered strain, which produces poly-(γ-glutamic acid) (γ-PGA) from raw extracted inulin of Jerusalem artichoke tubers; however, the underlying mechanisms remain unknown. To address this problem, we identified the inulin hydrolase in wild-type strain *B. amyloliquefaciens* NB.

**Results:**

The novel inulin hydrolase (CscA) was discovered from strain NB, with high inulinase activity (987.0 U/mg at 55 °C) and strong resistance at pH values between 8.0 and 11.0, suggesting the potential application of CscA in Jerusalem artichoke biorefinery. CscA exhibited a *k*_cat_/*K*_m_ of (6.93 ± 0.27) × 10^3^ for inulin; its enzymatic activity was stimulated by metal ions, like K^+^, Mn^2+^, or Ca^2+^. Similar to their role in glycoside hydrolase 32 family enzymes, the conserved Asp37, Asp161, and Glu215 residues of CscA contribute to its catalytic activity. Targeted disruption of *CscA* gene suppressed inulin utilization by strain NB. Overexpression of *CscA* significantly enhanced the γ-PGA generation by 19.2% through enhancement in inulin consumption.

**Conclusions:**

The inulin hydrolase CscA is critical for inulin metabolism in *B. amyloliquefaciens* and indicates potential application in Jerusalem artichoke biorefinery.

**Electronic supplementary material:**

The online version of this article (10.1186/s13068-019-1485-9) contains supplementary material, which is available to authorized users.

## Background

Inulin is a polydisperse fructan comprising linear chains of β-2,1-linked d-fructose molecules terminated by a glucose residue. It is found in tubers, roots, or leaves of many plants [1]. As a naturally inulin-rich candidate, Jerusalem artichoke (*Helianthus tuberosus*) has recently started drawing attention, as inulin can be used for production of many bio-based products, such as fructooligosaccharides, bioethanol, biodiesel, lactic acid, and other chemicals [2]. Inulin hydrolysis is the first step involved in Jerusalem artichoke biorefinery. Compared with the traditional techniques of acid hydrolysis, enzymatic simultaneous saccharification exhibits the advantages of alleviating substrate inhibition, simple processing, and environment friendly technique, which serves as an effective strategy in Jerusalem artichoke refining process [3]. However, the high price of inulinase (Novozym 960, $188 for 250 mL [product 16285; Sigma]) and the lack of efficient inulinase hydrolysis remain the main limiting factors for this process [4]. Inulinase is a member of glycosidase hydrolase family 32 (GH32) which specifically catalyzes the hydrolysis of fructans, similar to inulin. As an industrial food enzyme, inulinase can be classified into exo-inulinases (E.C. 3.8.1.80) or endo-inulinases (E.C. 3.2.1.7) based on the differential hydrolytic activity of the glycosidic bonds. An exo-inulinase usually functions by removing individual fructose moiety from the nonreducing end of inulin. Endo-inulinase can randomly break any β-2,1 glycosidic bond in inulin molecule, and can be used to produce inulotrioses (F3), inulotetraoses (F4), and other IOSs [5]. To date, many microorganisms have been reported to produce inulinase. As the predominant inulinase producers, yeast strains with high inulinase yield were screened. A newly isolated strain of *K. marxianus*, YS-1, was explored for its inulinase production and it was found that its maximum inulinase activity reached 55.4 U/mL within 60 h under the optimized conditions [6]. In addition, the purified exo-inulinase from *K. marxianus*, YS-1, showed the highest activity with an optimum pH of 5.5 and at the optimum temperature of 50 °C [7]. Gong et al. isolated the marine yeast, which secreted a large amount of inulinase, from the surface of a marine alga. After treating by using UV light and LiCl, an inulinase activity of 127.7 ± 0.6 U/mL was obtained in the liquid culture of the mutant *P. guilliermondii* M-30 using the RSM optimization. The crude inulinase exhibits optimal activity at pH 6.0 and 60 °C [8, 9]. Filamentous fungi are also inulinase chassis, which can secrete both exo- and endo-inulinases. Three exo-inulinases and two endo-inulinases were purified from the *Aspergillus ficuum* JNSP5-06 which were stable and exhibited optimum activity at the temperature range of 45–50 °C and pH of 4.5 for exo-inulinase and of 5.0 for endo-inulinase [10]. The molecular mass of the exo-inulinase purified from *Penicillium* sp. strain TN-88 was estimated to be 50.0 kDa and inulinase activity was optimum at pH 4.0 and 55 °C. An endo-inulinase, P-II, isolated from *Penicillium* sp., has a molecular weight of 68.0 kDa and its activity was highest at pH 5.2 and 50 °C [11]. As reported in previous studies, the optimum pH for most inulinases was in the range of 4.0–6.0, and the stability of this enzyme decreased significantly when the pH was increased to 6.0 leading to defects in refining process of fermentation at high pH [4]. Meanwhile, bacterial inulinase has attracted much attention due to its wider optimum pH range and stability at high temperature. A thermostable exo-inulinase was isolated from *Geobacillus stearothermophilus* KP1289 which had a molecular mass of 54.0 kDa and exhibited optimal activity at 60 °C [12]. Kang et al. reported that the *Arthrobacter* sp. S37 strain produced an endo-inulinase with a molecular weight of 75.0 kDa with an optimal pH and temperature of 7.5 and 50 °C, respectively. Under such conditions, the main products of inulin hydrolysis are inulotrioses, inulotetraoses, and inulopentaoses [13]. In addition, some other bacteria have been reported to produce inulinases, including *Xanthomonas* spp., *Bacillus* spp., *Clostridium* spp., *Staphylococcus* spp., and *Pseudomonas* spp. [14, 15]. Many studies have focused on fungi and yeasts for inulinase production, while there are only few studies concerning potent bacterial sources for inulinases due to low yield. It is of great significance to identify and excavate more efficient and potential bacterial candidates for adequate inulinase production for inulin biorefinery.

Poly-(γ-glutamic acid) (γ-PGA) is a bio-based homopolypeptide consisting of l- and d-glutamic acid units connected by γ-amide linkage [16]. Due to presence of several carboxylic groups on its side chain, γ-PGA exhibits properties of water absorption, moisture retention, and chelation, which has broad applications in fields of daily chemicals, food, medicine, environmental protection and agriculture [17]. Based on the response to exogenous glutamic acid, the γ-PGA-producing strains can be divided into two categories: the glutamic acid-dependent strain, which must ingest exogenous glutamic acid for adequate supply of substrate, such as *B. subtilis* NX-2 [18], and the glutamic acid-independent strain which possesses a non-essential precursor that has aroused extensive attention due to its economic γ-PGA fermentation. In a previous study, a novel strain named *Bacillus amyloliquefaciens* NX-2S was isolated from soil samples of Jerusalem artichoke tubers. The strain preferred to assimilate inulin as the sole carbon source and could convert the substrate inulin to γ-PGA directly via one-step fermentation reaction without the addition of exogenous glutamic acid [19]. Further increase in γ-PGA production was inhibited by the lack of the mechanisms of inulin metabolism in wild-strain *B. amyloliquefaciens*. Our study has for the first time identified and characterized the key enzyme responsible for inulin metabolism in a γ-PGA-producing strain *B. amyloliquefaciens* and identified a potential inulin hydrolysis candidate that can be used for efficient production of inulin-based bio-products.

## Methods

### Enzymes and chemicals

All restriction enzymes and the KOD DNA polymerase used for PCR were purchased from TaKaRa Bio, Inc. (China) and Toyobo (Osaka, Japan). Recombinant plasmids were generated using the ClonExpress™ II One Step Cloning Kit purchased from Vazyme Biotech Co., Ltd (Nanjing, China). Isopropyl-β-d-1-thiogalactopyranoside (IPTG) was obtained from Merck (Germany). Ampicillin, kanamycin, and chloramphenicol were purchased from Amresco (USA). Purified inulin was purchased from Xiya Reagent (CAS No: 9005-80-5, http://www.xiyashiji.com). Raw inulin extract was obtained from fresh Jerusalem artichoke tubers bought from Xuzhou Sheng Tong Food Co., Ltd (Xuzhou, China). All other chemicals were of analytical grade and were commercially available.

### Bacterial strains and plasmids

*Escherichia coli* strains, DH5α and BL21(DE3), were used for general cloning and protein expression, respectively. The plasmid pET-28a encoding a kanamycin resistance gene was used for protein expression. Lysogenic broth (LB) medium was used to cultivate *E. coli*. The plasmid pNX01 was used for CscA expression in *B. amyloliquefaciens. B. amyloliquefaciens* strain NB was used for γ-PGA fermentation. All bacterial strains and plasmids used in this study are listed in Table 1. The fermentation medium contained the following components (per liter): raw inulin extract (70 g), (NH_4_)_2_SO_4_ (5 g), K_2_HPO4·3H_2_O (20 g), KH_2_PO_4_ (1 g), MgSO_4_ (0.1 g), MnSO_4_·H_2_O (0.03 g); pH 7.5. *B. amyloliquefaciens* was inoculated into a 500-mL flask containing 80 mL of the above-described seed medium and cultivated at 32 °C for 12 h with shaking at 200 rpm. Then, the seed culture was inoculated into Erlenmeyer flasks or bioreactors (6% of the total volume) for γ-PGA production. When required, the media were supplemented with ampicillin (Amp; 100 μg/mL) or kanamycin (Kana; 25 μg/mL) for *E. coli* and spectinomycin (Spec; 25 μg/mL) or chloromycetin (Cl; 6 μg/mL) for *B. amyloliquefaciens*.

**Table 1:** Strains and plasmids used in this study

Strains or plasmids	Relevant properties	Source
Strains		
*E. coli* DH5α	F^−^, φ80d*lac*ZΔM1, Δ(*lacZYA*-*argF*) U169, *deoR*, *recA*1, *endA1*, *hsdR*17 (rk^−^, mk^+^), *phoA*, *supE*44, λ^−^*thi*-1, *gyrA*96, *relA*1	This lab
*E. coli* GM2163	F^−^, *ara*-14 *leuB*6 *thi*-1 *fhu*A31 *lacY*1 *tsx*-78 *galK*2 *galT*22 *supE*44 *hisG*4 *rpsL* 136 (*Str*^r^) *xyl*-5 *mtl*-1 *dam*13::Tn9 (Cam^r^) *dcm*-6 *mcrB*1 *hsdR*2 *mcrA*	This lab
*E. coli* BL21(DE3)	F-, *ompT hsdSB* (rB^−^ mB^−^) *gal dcm* (DE3)	This lab
*B. amyloliquefaciens* NB	NX-2S derivate, *BamHI*::P_HpaII_-*pgsR*	This lab
*B. amyloliquefaciens* NBΔC	NBΔ*CscA*	This study
*B. amyloliquefaciens* NBΔC-C	NBΔ*CscA* containing pNX-*CscA*	This study
*B. amyloliquefaciens* NB-C	NB containing pNX-*CscA*	This study
Plasmids		
pNX01	*E. coli* and *B. amyloliquefaciens* expression vector; *Amp*^*R*^, *Cm*^*R*^	This lab
pNX01-*CscA*	pNX01 carrying *CscA* gene from *B. amyloliquefaciens* NB used for overexpression of the *CscA* gene	This study
pDR-pheS*	*E. coli* and *B. amyloliquefaciens* knockout vector; *Amp*^*R*^, *Spec*; pDR with P_P43_-pheS* cassette	This lab
pDR-pheS*-*CscA’*	pDR-pheS* carrying the homology arm of *CscA* gene used for deletion of the *CscA* gene	This study

### Cloning and expression of predicted genes involved in inulin hydrolysis

To obtain the *sacC* gene (Genebank: WP_014472414.1) from strain *B. amyloliquefaciens* NB, two primers, S1-F and S1-R, was designed to amplify the *sacC* sequence using PCR. Genomic DNA was isolated from *B. amyloliquefaciens* NB using a Takara Bacterial Genomic DNA Extraction Kit (Takara, China). The amplified 1.4-kb DNA fragment was cloned into the pET-28a vector by *Bam*HI and *Xho*I using One Step Cloning Kit and transformed into *E. coli* DH5α competent cells. The constructed vector, named pET28a-*sacC*, was validated by sequencing and transformed into *E. coli* BL21 (DE3). To overexpress *CscA* (Genebank: WP_013353709.1) in *E. coli*, the *CscA* fragment was ligated to pET-28a vector which was digested using *Bam*HI and *Xho*I. The resulting plasmid was designated as pET-*CscA* and the cells were transformed into *E. coli* BL21 (DE3) for expression.

### Characterization of the inulin hydrolase (CscA)

Recombinant *E. coli* BL21 (DE3) cells harboring the plasmid pET-*CscA* were grown at 37 °C in LB medium containing kanamycin (25 μg/mL) until the OD_600nm_ reached 0.5. Then, IPTG was added at concentration of 1 mM and the cells were incubated at 25 °C for further 12 h. After induction, the cells were harvested by centrifugation at 8000*g* for 10 min and washed twice with 50 mM phosphate buffer (pH 6.5). After sonication, the homogenate were centrifuged to remove the cell debris and the filtrate was recovered through a 0.2 m filter was loaded on an Ni–NTA resin column equilibrated with equilibration buffer (300 mM NaCl, 50 mM NaH_2_PO_4_; pH 8.0). The column was then washed with the same buffer supplemented with 10 mM imidazole, and a gradient of imidazole (from 50 to 250 mM) was employed to elute the recombinant protein. The fraction with inulinase activity was collected and concentrated in an Amicon Ultra-15 centrifugal filter unit (10-kDa cutoff). Expressed and purified enzymes were analyzed by SDS-PAGE analysis.

### Effect of temperature and pH on enzyme activity and stability

To identify the optimum temperature for CscA, assays were measured over the temperature range of 25 °C to 80 °C, at pH 6.5. Three buffer systems (Na_2_HPO_4_–citrate acid/phosphate/glycine–NaOH) were used to determine the pH for optimum enzyme activity at 55 °C. All buffers were used at a concentration of 1/15 M. The thermal stability of CscA was investigated by incubating the enzyme at the temperature range from 30 to 75 °C in PBS buffer (pH 6.5). Similarly, the enzyme solution was incubated at various pH values (4.0 to 11.0) at 55 °C for 1 h to study the effect of pH on enzyme stability.

### Effect of various metal ions on enzyme activity

For determination of effects of different metal ions on inulinase activity, the purified CscA was dialyzed against PBS buffer (1/15 M, pH 7.5) containing 10 mM EDTA overnight at 4 °C. Then, the enzyme was dialyzed against PBS buffer (1/15 M, pH 7.5) to remove EDTA. Subsequently, the enzyme concentration was concentrated to the level as before dialysis. And the acquired enzyme was assessed in the presence of different metal ions (ZnCl_2_, MnCl_2_, CaCl_2_, CoCl_2_, KCl, CuCl_2_, AlCl_3_, and NaCl) with a final concentration of 1 mM or 10 mM. Then, the enzyme activities after incubation were determined under assay conditions.

### Determination of substrate specificity and kinetic parameters

For determination of the substrate specificity, purified CscA enzyme was incubated under standard reaction conditions with different substrates (50 mM of inulin, sucrose, raffinose, stachyose, fructooligosaccharide, and soluble starch). Kinetic parameters of the enzyme were determined for different substrates in 50 mM PBS buffer (pH 7.5). The substrates were dissolved in PBS buffer (pH 7.5) and incubated with purified CscA enzyme at 55 °C for 20 min. Kinetic parameters, such as the Michaelis–Menten constant *K*_m_ (mM) and maximum reaction velocity *V*_max_ (U/mg protein) for substrates, were obtained using Lineweaver–Burk plots. All assays were performed in triplicates at least two separate times.

### Site-directed mutagenesis of inulin hydrolase (CscA)

To construct site-directed mutants of *CscA* gene, three conserved amino acid residues (GAT for Asp37, GAC for Asp161, and GAA for Glu215) were substituted by GCG for Ala, by overlap extension PCR method [20]. The mutant of D37A was constructed as follows: The plasmid pET28a-*CscA* was used as template, and the forward primer, pETCSCA-F, and mutant primer, D37A-1, were used for PCR I amplification (the product contained upstream fragments of the mutant site). In addition, the reverse side primer, pETCSCA-R, and mutant primer, D37A-2, were used for PCR II amplification. The products were separated by agarose gel electrophoresis and recovered from the gel. Using mixtures of PCR I and PCR II as template, PCR III was done with positive and reverse primers, pETCSCA-F and pETCSCA-R, respectively. The PCR reaction mixture contained 2 µL template PCR I, 2 µL template PCR II, 2 µL of each primer, 25 µL KOD mix (G497-dye, Abcam, China), and 17 µL double distilled H_2_O. The thermal cycling parameters consisted of an initial denaturation step at 94 °C for 10 min, followed by 30 cycles of 94 °C for 40 s, 55 °C for 30 s, and 72 °C for 2 min, and a final elongation step after the final cycle at 72 °C for 10 min. After gel purification, 1.5-kb fragment was introduced into *Bam*HI–*Xho*I cleaved pET28a vector using the ClonExpress II One Step Cloning Kit. The generated plasmid was introduced into *E. coli* BL21 (DE3) for D37A mutant expression. D161A and E215A mutants were constructed as described above.

### Inulin hydrolase knockout and complementation

To inactivate the *CscA* gene in the chromosome of wild-strain *B. amyloliquefaciens*, an internal 800-bp fragment of the *cscA*’ gene was cloned into the temperature-sensitive plasmid, pDR-pheS* digested with *EcoR*I and *Xho*I. The resulting plasmid pDR-pheS*-*cscA*’ could replicate normally at 30 °C, but not at high temperature, integrating the plasmid into the chromosome. The pDR-pheS*-*cscA*’ plasmid was extracted from Dam methylation-deficient strains of *E. coli* GM2163, and then, transformed into *B. amyloliquefaciens* according to a modified high osmolarity electroporation method [21]. After sequence validation using primers pDR-F and pDR-F, the positive clones containing the pDR-pheS*-*cscA*’ plasmid were cultivated at 30 °C with Spec, and the transformants were transferred to 42 °C with Spec to facilitate plasmid integration. The crossover integrants were further amplified by PCR using primers CSCA-OUT-F and CSCA-OUT-R. In the complementation assay, a newly constructed plasmid, pNX01, based on a native replicon from endogenous plasmid, p2Sip, was used for CscA expression, under the control of strong promoter P_p43_ in *B. amyloliquefaciens.* The inulin hydrolase gene (*CscA*) was PCR amplified from the genomic DNA of *B. amyloliquefaciens* using primes pNCSCA-F/pNCSCA-R and cloned into the plasmid, pNX01, digested with *Spe*I and *Not*I. The resulting plasmid, pNX-*CscA*, was then electro-transferred into the *B. amyloliquefaciens* strain. All the primers used in this study are listed in Table 2.

**Table 2:** Primers and their sequences used for PCR in this study

Primers	Sequences (5′–3′)
pETSACC-F	CAGCAAATGGGTCGC*GGATCC*ATGGCACCAAAATGGCCC
pETSACC-R	GTGGTGGTGGTGGTG*CTCGAG*CTAAGGATGAATCGAACCGAGAT
pETCSCA-F	CAGCAAATGGGTCGC*GGATCC*ATGGATAGAATTCAGCAGGCGG
pETCSCA-R	GTGGTGGTGGTGGTG*CTCGAG*TCACTTTTTTTCCAAATGTCCTTTAC
D37A-1	CTTAAACTGAATAAGTCCGTTCGG*CGC*ATTGATCCAATTCGCCCGGG
D37A-2	TTCAAAGAAATCAGGACA*CGC*CCACATATAGCCGAGATG
D161A-1	TTCAAAGAAATCAGGACA*CGC*CCACATATAGCCGAGATG
D161A-2	GGTCATCTCGGCTATATGTGG*GCG*TGTCCTGATTTCTTTGAATTA
E215A-1	GCGATGCTTCCACACTTTCGG*CGC*GCGAAAATGGCGGGAGCT
E215A-1	AGCTCCCGCCATTTTCGC*GCG*CCGAAAGTGTGGAAGCAT
pNXCSCA-F	AAAAGGAGCGATTTA*GGTACC*ATGGATAGAATTCAGCAGGCGG
pNXCSCA-R	TGTTTTTTTATTACC*CATATG*TCACTTTTTTTCCAAATGTCCTTTAC
pDRCSCA’-F	TAGATAGCGCATGCT*GAATTC*CCGCCGGAAGACAGCTCC
pDRCSCA’-R	ACATACTTTAAAAAT*CTCGAG*CGCACTCTTCTCACACCGTCT
CSCAOUT-F	CCATGACCGAGGATTTGCGGACAATG
CSCAOUT-R	TGGACCGCTCCTGCAGTACAACGTCT

### Analytical methods

The inulinase activity was assayed using the method described by Li et al. with slight modifications [4]. A reaction mixture containing 0.2 mL of the enzyme, 0.8 mL of phosphate-buffered saline (pH 7.5), and 2% inulin was incubated at 50 °C for 20 min. Then, the enzyme was inactivated by incubating the reaction mixture at 100 °C for 10 min. The reducing sugars in the reaction mixture were measured using the 3,5-dinitrosalicylic acid method. One unit (U) of levanase was defined as the amount of enzyme that yielded 1 mol of reducing sugars per min under the assay conditions used in this study. The specific activity of levanase was defined as the units of enzyme (U) divided by the amount of enzyme (mg). The cell density was monitored by measuring the optical density of the deposit at 600 nm using an ultraviolet spectrophotometer (Spectrumlab 752 s; LengGuang Tech, China). The cell density was determined by measuring the absorbance of the culture broth at 660 nm using an ultraviolet spectrophotometer (752 N, Shanghai, China). After dilution, the culture broth was centrifuged at 8000*g* for 20 min, and the cell precipitate was collected, washed twice with distilled water, and dried at 105 °C until constant weight for dry cell weight (DCW) measurement. The γ-PGA concentration and molecular weight were determined by gel permeation chromatography (GPC) using an RI-10 refractive index detector and a SuperposeTM 6 column (Shimadzu Co.). Inulin or sucrose consumption was evaluated using HPLC method as described previously [19].

## Results

### Prediction and identification of the inulin hydrolase in *B. amyloliquefacien*s

**Fig. 1:**
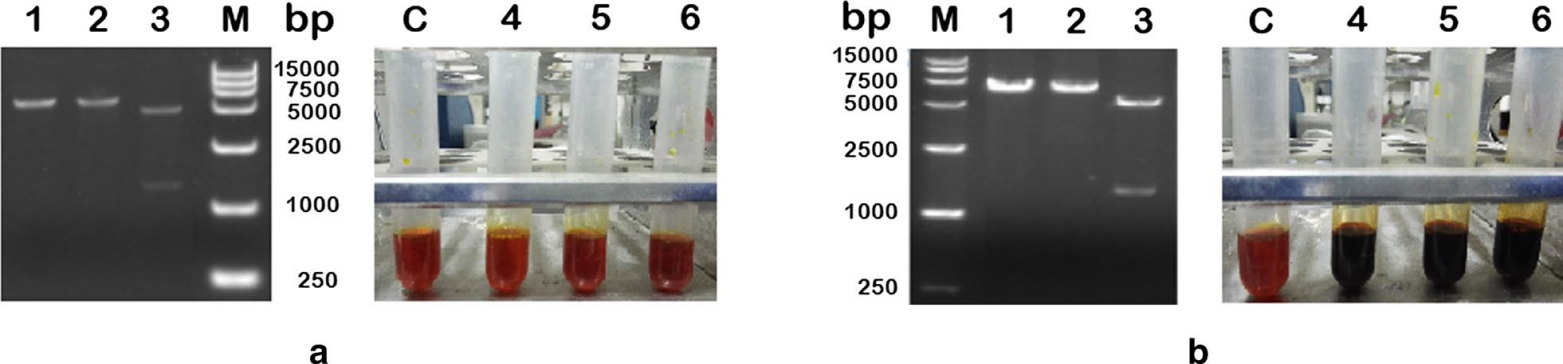
Heterologous expression and functional validation of the putative inulin hydrolysis encoding genes from *B. amyloliquefaciens* in *Escherichia coli*. **a** Restriction analysis of recombinant plasmid pET28a-*sacC*. Lanes 1 and 2: pET28a-*sacC* digested with *Bam*HI or *Xho*I; lane 3: pET28a-*sacC* digested with *Bam*HI and *Xho*I; and lane M: DL15,000 marker. Inulinase activity was determined by DNS method. Lane C: the control crude extract and lanes 4, 5, and 6: inulinase activity detected at 30, 45, and 55 °C, respectively. **b** Restriction analysis of recombinant plasmid pET28a-*CscA*. Lanes 1 and 2: pET28a-*CscA* digested with *Bam*HI or *Xho*I; lane 3, pET28a-*CscA* digested with *Bam*HI and *Xho*I; and lane M: DL15,000 marker. Inulinase activity was determined by DNS method. Lane C: the control crude extract and lane 4, 5, and 6, inulinase activity detected at 30, 45, and 55 °C, respectively

Due to growing number of studies on inulin which serves as a renewable carbohydrate raw material for biotechnology, enzymes involved in the hydrolysis of polyfructans have developed great interest [22]. *Bacillus* spp. has been confirmed as a chassis source of bacterial inulinases. An exo-inulinase was cloned and characterized from *Paenibacillus polymyxa* ZJ-9. The purified enzyme showed maximum activity at 25 °C and pH 6.0 [23]. Liu et al. characterized a thermo-stable endo-inulinase from a novel strain *Bacillus smithii* T7, which was stable and functioned optimally at pH 4.5 and at temperature of 70 °C [24]. *B. amyloliquefaciens* NB is a suitable microorganism isolated previously to conduct one-step γ-PGA fermentation from inulin substrate in absence of glutamate. However, genetic mechanisms of inulin metabolism in such strains are unknown. This is mainly because no genes encoding inulinase have been previously found in *B. amyloliquefaciens*. In another group of *B. subtilis* with high affinity to *B. amyloliquefaciens*, the levanase (*SacC*) in sucrose operon has been reported to be active against various substrates, such as levan, inulin, and sucrose [22]. Inhibition of *SacC* gene suppressed the inulinase activity in *Bacillus subtilis* WB800, which suggested the levanase was involved in inulin hydrolysis [25]. Based on these findings, to identity whether the levanase in strain *B. amyloliquefaciens* NB could also hydrolyze inulin, the levanase gene, *sacC*, was cloned into *Bam*HI–*Xho*I cleaved pET28a vector to generate the expression plasmid pET28a-*sacC*, which was expressed in *E. coli* under the control of T7 promoter. However, a crude extract of induced *E. coli* BL21(DE3)/(pET28a-*sacC*) cells showed no detectable activity on inulin as shown in Fig. 1a. The results indicated that levanase was not responsible for inulin metabolism in wild strain *B. amyloliquefaciens.*

**Fig. 2:**
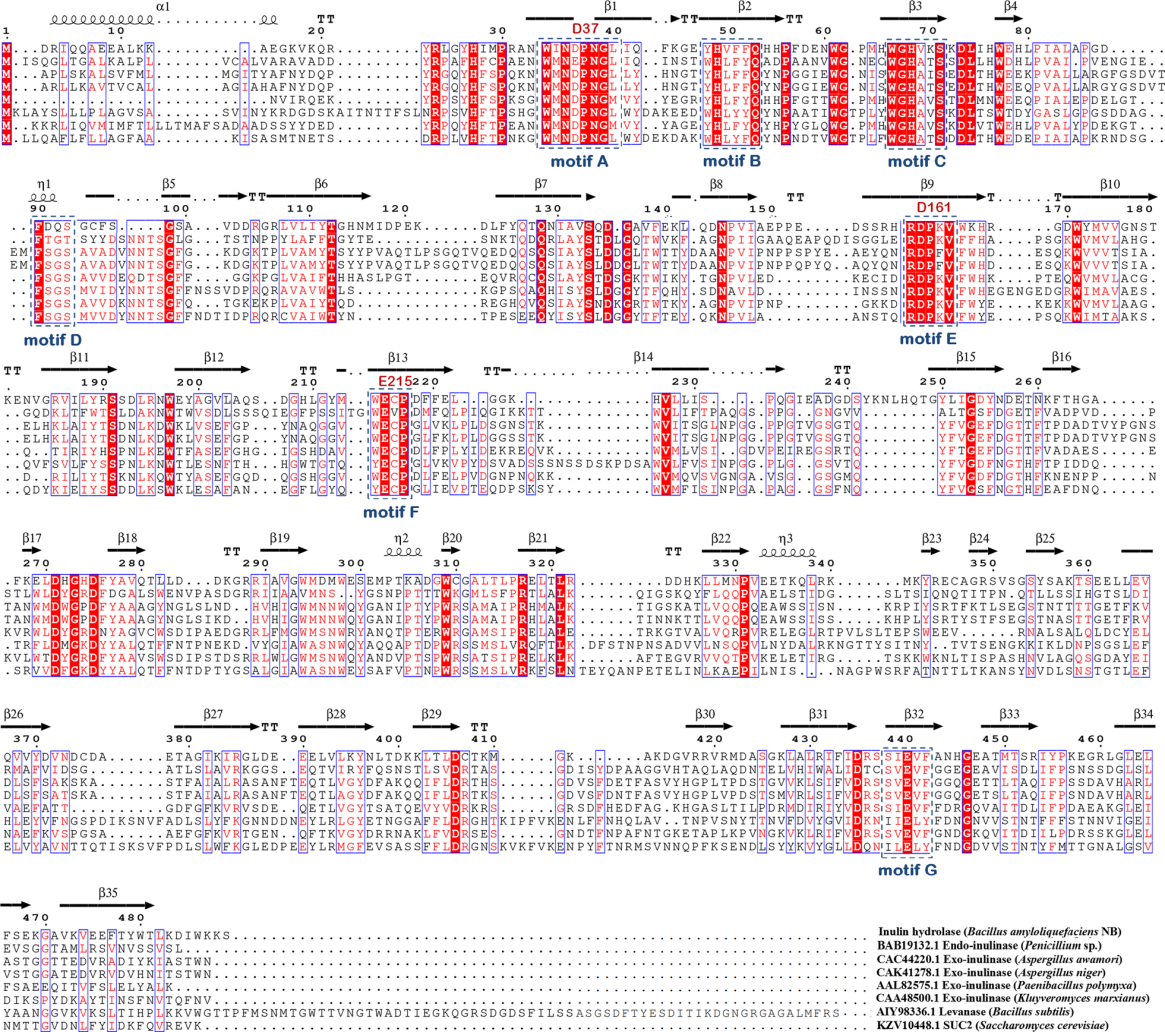
Multiple-sequence alignment of inulinases, levanase, and inulin hydrolase CscA of *B. amyloliquefaciens* NB. The alignment was performed using CLUSTALW and the image was prepared using ESPript. Secondary structure elements are also shown

As a member of glycoside hydrolase 32 family, there are seven conserved motifs containing WMN(E/D)PNG [A], WHLFFQ [B], WGHATS [C], F(T/S)G(T/S) [D], RDPKV [E], E(V/C)P [F], and SVEVF [G] in inulinases or levanases [26]. Genome analysis of *B. amyloliquefaciens* revealed a putative gene named, *CscA*, consisting of all conserved domains of inulinases and was speculated to have similar functions and role in inulin hydrolysis. Based on the genome sequence of *B. amyloliquefaciens,* the *CscA* gene structure contains an ORF of 1470 bp long that encodes 489 amino acids and does not possess a classical bacterial signal peptide as analyzed by SignalP 4.1 Server. Comparing the amino acid sequences with those of other inulinases or levanase from various sources, the hypothetical protein sequence showed a very low homology with most of the other sequences as seen from multiple-sequence alignment (Fig. 2). The protein, CscA, showed 25%, 26%, and 22% similarity to exo-inulinase of *Aspergillus awamori*, *Aspergillus niger*, and *Kluyveromyces marxianus*, respectively. In addition, the purified CscA sequence shared 23% identity with *Penicillium* sp. endo-inulinase, 21% identity with *Saccharomyces cerevisiae* invertase, 24% identity with *B. subtilis* levanase, and 27% identity with exo-inulinase from *Paenibacillus polymyxa*. The results showed that the enzyme, CscA, produced from *Bacillus amyloliquefaciens* may be a novel enzyme involved in inulin hydrolysis. To further verify the inulinase activity of the hypothetical protein sequence, the *CscA* gene was inserted into the vector pET28a and the plasmid pET28a-*CscA* was then transformed into *E. coli* BL21, followed by selection of positive colonies, as described previously in “Methods” section. Interestingly, high inulinase activity (43.78 ± 2.7 U/mg) was detected in the crude extract of cells induced with IPTG, and no inulinase activity was detected in the control extract (Fig. 1b). These results demonstrated that the identified novel *CscA* is involved in inulin metabolism in *B. amyloliquefaciens*.

### Characterization of the inulin hydrolase (CscA) isolated from *B. amyloliquefaciens* NB

#### Optimum temperature and thermostability

**Fig. 3:**
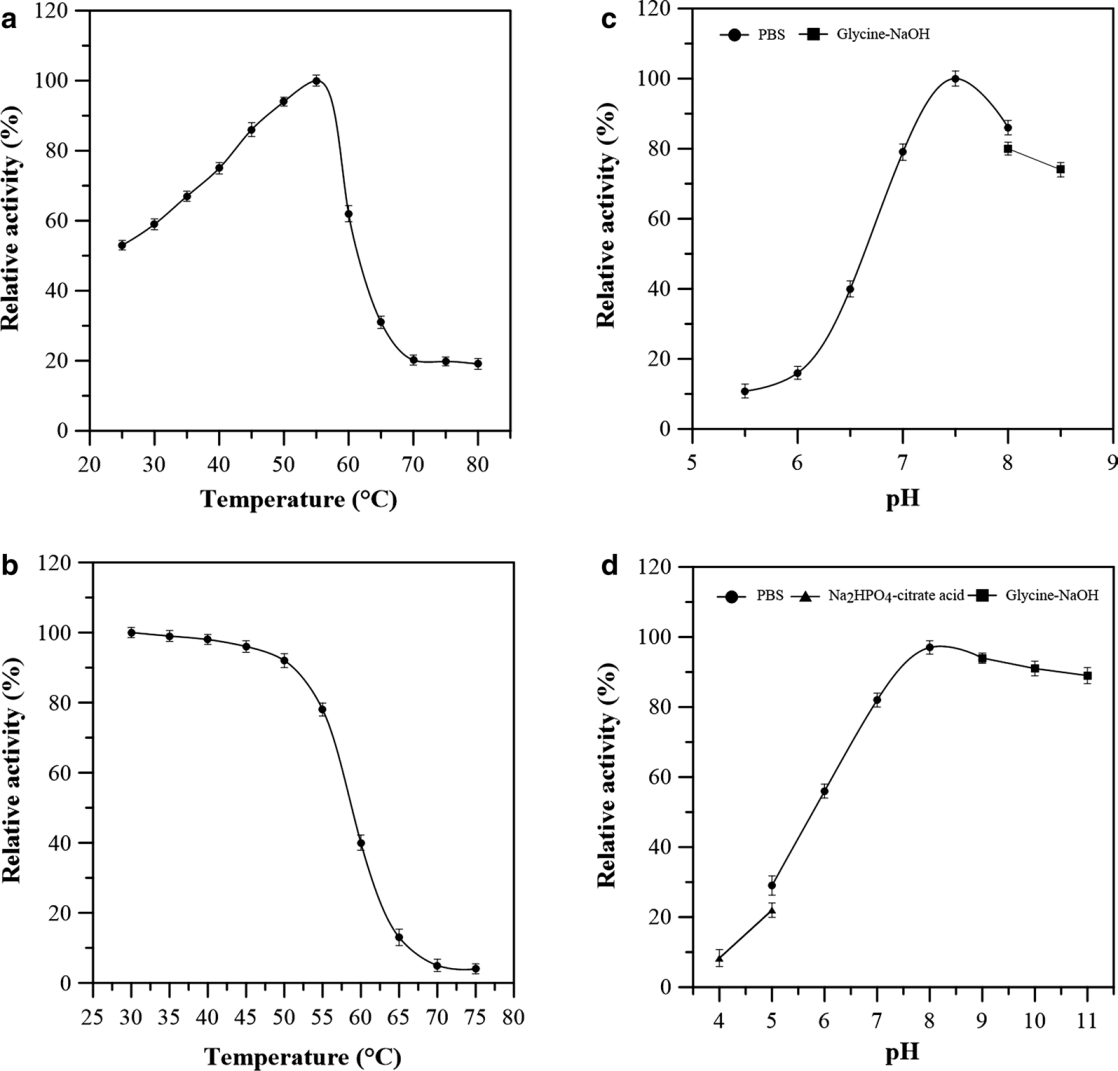
Characterization of the inulin hydrolase (CscA) from *B. amyloliquefaciens* NB. **a** Optimum temperature; **b** temperature stability; **c** optimum pH; and **d** pH stability. The buffer used for the thermal stability assay was PBS. The buffers used for the pH stability assay were as follows: pH 3–5, Na_2_HPO_4_–citrate acid buffer; pH 5–8, PBS buffer; and pH 8–10, glycine–NaOH buffer

The temperature range for optimum inulinase activity of the purified inulin hydrolase, CscA, was found to be in between 25 and 80 °C. The maximum activity of the purified CscA was observed at 55 °C (Fig. 3a). As shown in Fig. 3b, CscA exhibited good thermostability at temperatures below 50 °C, whereas 78% activity was still detected at 60 °C after treatment for more than 1 h. In addition, CscA was inactivated rapidly when treated to temperature of 70 °C for 1 h. The results showed that optimum temperature of the purified CscA was almost similar to that of most reported inulinases [15]. Although inulin, as a substrate, can be metabolized in SSF with the help of most thermophilic inulinases, which enhance inulin solubilization and minimizes the risks of microbial contamination, the use of mesophilic inulinases for the one-step fermentation of inulin is desirable because microbial fermentation processes are usually conducted under the conditions of 25 °C to 37 °C [23]. Generally, due to the thermostability of CscA in range 30–50 °C, the CscA with high inulinase activity offered several advantages in the vast majority of inulin-based microbial fermentation processes.

#### pH optimum and stability

Effects of pH on the inulinase activity of the purified CscA from *B. amyloliquefaciens* NB is shown in Fig. 3c. The optimum pH range for inulinase activity was measured by assaying the purified CscA at pH values between 5.5 and 8.5. Maximum inulinase activity was obtained at pH 7.5 (Fig. 3d). However, the optimal pH range of the inulinases isolated from fungi and yeasts is 4.5–6.0 [15]. In contrast, when the purified CscA was incubated at pH lower than 6.0 for 1 h, inulinase activity decreased rapidly. In addition, the CscA was stable at pH values between 8.0 and 11.0, suggesting that the novel enzyme exhibited strong resistance under the alkaline conditions.

#### Effects of metal ions on enzyme activity

A range of metal ions were examined to determine their effect on the purified CscA using inulin as the substrate. As shown in Table 3, K^+^, and Ca^2+^ stimulated the activity of the purified enzyme, similar to previous reported inulinases isolated from marine yeast [27]. In addition, it was found that Mn^2+^ also enhanced enzymatic activity of the CscA, whereas Co^2+^, Zn^2+^ and Cu^2+^ strongly inhibited its activity. Other ions, such as Al^3+^ and Na^+^, had no significant influence on inulin-hydrolyzing activity of CscA.

**Table 3:** Effects of different metal ions on the activity of CscA

Metal ions	Relative activity (%)	
	1 mM	10 mM
None^a^	100 ± 1	100 ± 2
EDTA^b^	99 ± 1	97 ± 1
ZnCl_2_	83 ± 1	76 ± 1
MnCl_2_	125 ± 1	131 ± 2
CaCl_2_	114 ± 1	117 ± 1
CoCl_2_	92 ± 1	84 ± 1
KCl	109 ± 2	115 ± 2
CuCl_2_	43 ± 2	–
AlCl_3_	102 ± 1	97 ± 2
NaCl	97 ± 1	92 ± 1

The inulinase activity of EDTA-treated CscA was assayed in PBS buffer (1/15 M, pH 7.5) at 55 °C for 20 min after incubating of 1 mM or 10 mM various metal ions. Each value represents the mean of triplicate measurements and varied from the mean by not more than 10%

^a^The activity of purified CscA enzyme without EDTA treatment and metal ions addition was set as 100%

^b^The activity of purified CscA enzyme after EDTA treatment but without metal ions addition

#### Enzyme kinetics and substrate specificity

In *K. marxianus*, inulinase interacts with sucrose, raffinose, stachyose, and inulin as substrates [28]. The levanase purified from *B. subtilis* mainly hydrolyzed inulin, sucrose, and to a small extent, raffinose, whereas it did not hydrolyze melezitose, stachyose, cellobiose, maltose, and lactose [22]. The inulin hydrolase produced by *B. amyloliquefaciens* NB was investigated in order to verify its specificity for various substrates. Similar to other inulinases or levanase, the purified CscA enzyme can hydrolyze inulin, sucrose, raffinose, stachyose, fructooligosaccharide, and soluble starch suggesting the CscA has a broad spectrum of substrate recognition. The kinetic parameters are summarized in Table 4. All parameters were calculated with enzyme concentrations determined using the micromethod of Bio-Rad; a molecular mass of 65.0 kDa was determined for CscA. The results showed that the kinetic constant values for apparent *K*_m_ and *V*_max_ of purified enzyme were 8.35 ± 0.04 mM and (9.92 ± 0.31) × 10^2^ µmol min^−1^ mg^−1^, 4.33 ± 0.03 mM and (1.76 ± 0.05) × 10^3^ µmol min^−1^ mg^−1^, and 10.22 ± 0.06 mM, (9.68 ± 0.32) × 10^2^ µmol min^−1^ mg^−1^ toward inulin, sucrose, and fructooligosaccharide (Table 4), respectively. In addition, the *K*_m_ and *V*_max_ values of the CscA toward raffinose and stachyose were 12.25 ± 0.07 mM and (7.24 ± 0.11) × 10^2^ µmol min^−1^ mg^−1^, 19.08 ± 0.09 mM and (6.87 ± 0.29) × 10^2^ µmol min^−1^ mg^−1^, respectively. Relatively lower *K*_m_ (4.33 mM) and higher catalytic efficiency, *k*_cat_/*K*_m_ ((6.93 ± 0.27) × 10^3^ min^−1^ mM^−1^), of the purified CscA for sucrose demonstrated its greater affinity of sucrose rather than inulin, unlike the exo-inulinase from *Penicillium janczewskii*, which exhibited a *K*_m_ value for sucrose that was twofold lower than that for inulin [29]. Inulinase can specifically hydrolyze beta-2,1-fructan, which means that it can hydrolyze sucrose to fructose and glucose at the same time as inulin. However, there are more specific sucrase enzymes (EC 3.2 1. 26) in the hydrolysis of sucrose. Inulinase and sucrase belong to beta-fructofuran glycosidase. They usually show the activity of two enzymes, but the enzymatic activity is different. By determining the substrate specificity of the purified CscA, we found that the affinity of the enzyme CscA to sucrose was much higher than that of inulin, suggesting that the enzyme might be more inclined to sucrase or invertase. By the sequence aliment with invertase or invertase showed that the enzyme CscA had low homology with other invertase but contained conserved inulinase sequences which other invertase did not have (Additional file 1: Figure S1). Therefore, it also shows that the enzyme has two common catalytic centers, but the binding sites of inulin or sucrose are different. This phenomenon is accompanied by the action of sucrase SUC2 from *S. cerevisiae* [30]. The results showed that inulin degraded in the absence of intermediate inulin oligosaccharides, indicating that CscA was more likely to be an exo-type inulin hydrolase (Additional file 1: Figure S2).

**Table 4:** Substrate specificity of purified CscA of *B. amyloliquefaciens* NB

Substrate^a^	*V*_max_ (μmol min^−1^ mg^−1^)	*K*_m_ (mM)	*k*_cat_/*K*_m_ (min^−1^ mM^−1^)
Inulin	(9.92 ± 0.31) × 10^2^	8.35 ± 0.04	(6.93 ± 0.27) × 10^3^
Sucrose	(1.76 ± 0.05) × 10^3^	4.33 ± 0.03	(2.39 ± 0.12) × 10^4^
Raffinose	(7.24 ± 0.11) × 10^2^	12.25 ± 0.07	(3.45 ± 0.08) × 10^3^
Stachyose	(6.87 ± 0.29) × 10^2^	19.08 ± 0.09	(2.19 ± 0.04) × 10^3^
Fructooligosaccharide	(9.68 ± 0.32) × 10^2^	10.22 ± 0.06	(5.84 ± 0.05) × 10^3^
Soluble starch	–	–	–

^a^All substrates were dissolved or suspended in PBS buffer

#### Determination of catalytic sites of CscA involved in inulin hydrolysis

**Fig. 4:**
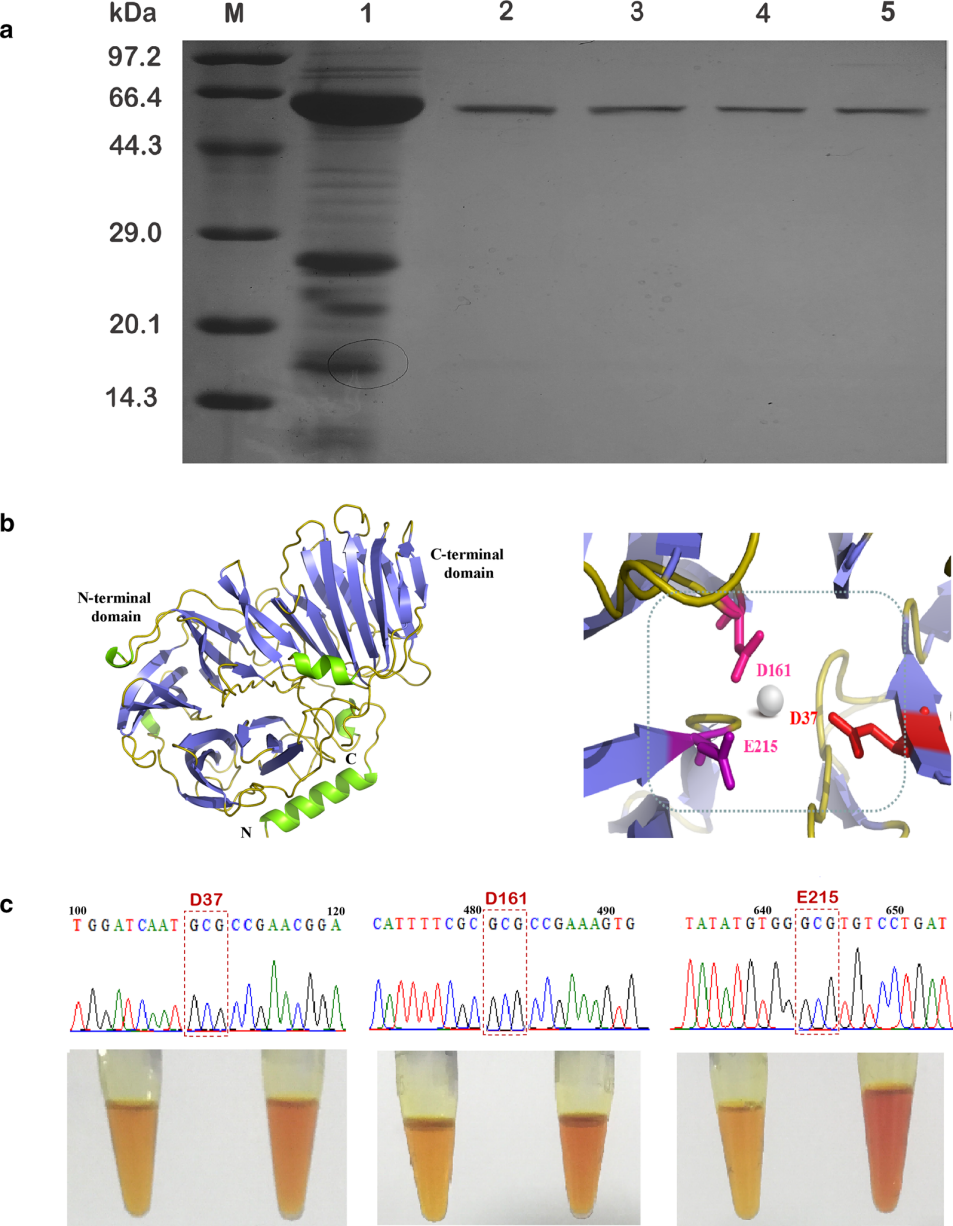
Determination of putative catalytic sites of the CscA. **a** SDS-PAGE analysis of the purified unmutated CscA enzyme and Asp37, Asp161, and Glu215 mutant enzymes. Lane M: premixed protein marker (low); lane 2: purified CscA enzyme; lane 3: D37A; lane 4: D161A; lane 5: E215A. **b** The secondary structure and putative catalytic sites of the CscA, superposed to *A. awamori* exo-inulinase (PDB entry 1Y9G). **c** Inulinase activity assay of mutant inulinase hydrolases

According to amino acid sequence alignment with other inulinases or levanase, WMN(D/E)PN, RDP, and E(C/V)P domains were conserved in the primary structure of CscA enzyme (Fig. 2). Reddy’s study, centered on the yeast invertase activity, revealed that Asp23 and Glu204 in the WMN(D/E)PN domain and E(C/V) P domain, respectively, were catalytic residues of the enzyme, which played nucleophilic and acid–base catalytic roles, respectively [31]. Crystal structure of *T. maritima* invertase, 1UYP, and *A. awamori* exo-inulinase, 1y4wA, confirmed this classical acid–base catalytic mechanism, and Asp138 in 1UYP and Asp189 in 1y4wA (corresponding to D in RDP sequence) act as binding substrates [28, 32]. In order to study the essential amino acids associated with the activity of inulinase, the site-specific mutations of Asp37, Asp161, and Glu215 in the conserved domains of CscA were selected to determine the catalytic residues involved in the recombinant fructanase activity (Fig. 4). Table 5 shows the kinetic parameters of the wild-type and various mutant CscAs. The loss of the Asp37 or Glu215 residues resulted in highly decreased enzymatic efficiency. The *K*_m_ values of D37 and E215 mutant CscA were similar to that of wild-type CscA, suggesting that these residues elicited a nucleophilic and an acid/base catalytic activity rather than being involved in substrate recognition. However, the efficiencies of these mutant enzymes (*k*_cat_/*K*_m_) were 0.06% and 0.04%, respectively, compared to that of the wild-type enzyme. As a result, these predicted residues with carboxylate groups were speculated to play an important role in inulinase activity which was consistent with previous reports on *Arthrobacter* sp. S37 inulinase and yeast invertase [31, 33]. Glu215 in the RDP motif was substituted by Ala, which led to increase in the *K*_m_ value of the E215A mutant from 8.35 ± 0.04 to 9.74 ± 0.12 mM, while the *k*_cat_ value was decreased tenfold. The results suggested the importance of Glu215 residue of the RDP motif in the catalytic function of the CscA. Based on these findings, we determined the roles of the conserved Asp37, Asp161, and Glu215 residues in the catalytic activity of the CscA enzyme and laid a theoretical basis for further improving enzymatic activity.

**Table 5:** Kinetic parameters of the wild-type and the CscA mutations

	*K*_cat_ (min^−1^)	*K*_m_ (mM)	*K*_cat_/*K*_m_ (min^−1^ mM^−1^)	Relative enzyme efficiency (%)
Wild-type	57,800 ± 0.38	8.35 ± 0.04	6930 ± 0.27	100
D37A	3.4 ± 0.05	8.29 ± 0.05	4.1 ± 0.12	0.06
D161A	2.4 ± 0.11	8.32 ± 0.08	3.1 ± 0.08	0.04
E215A	5800 ± 0.09	9.74 ± 0.12	595 ± 0.14	8.6

#### Effect of cscA gene for inulin metabolism for γ-PGA production in *Bacillus amyloliquefaciens*

**Fig. 5:**
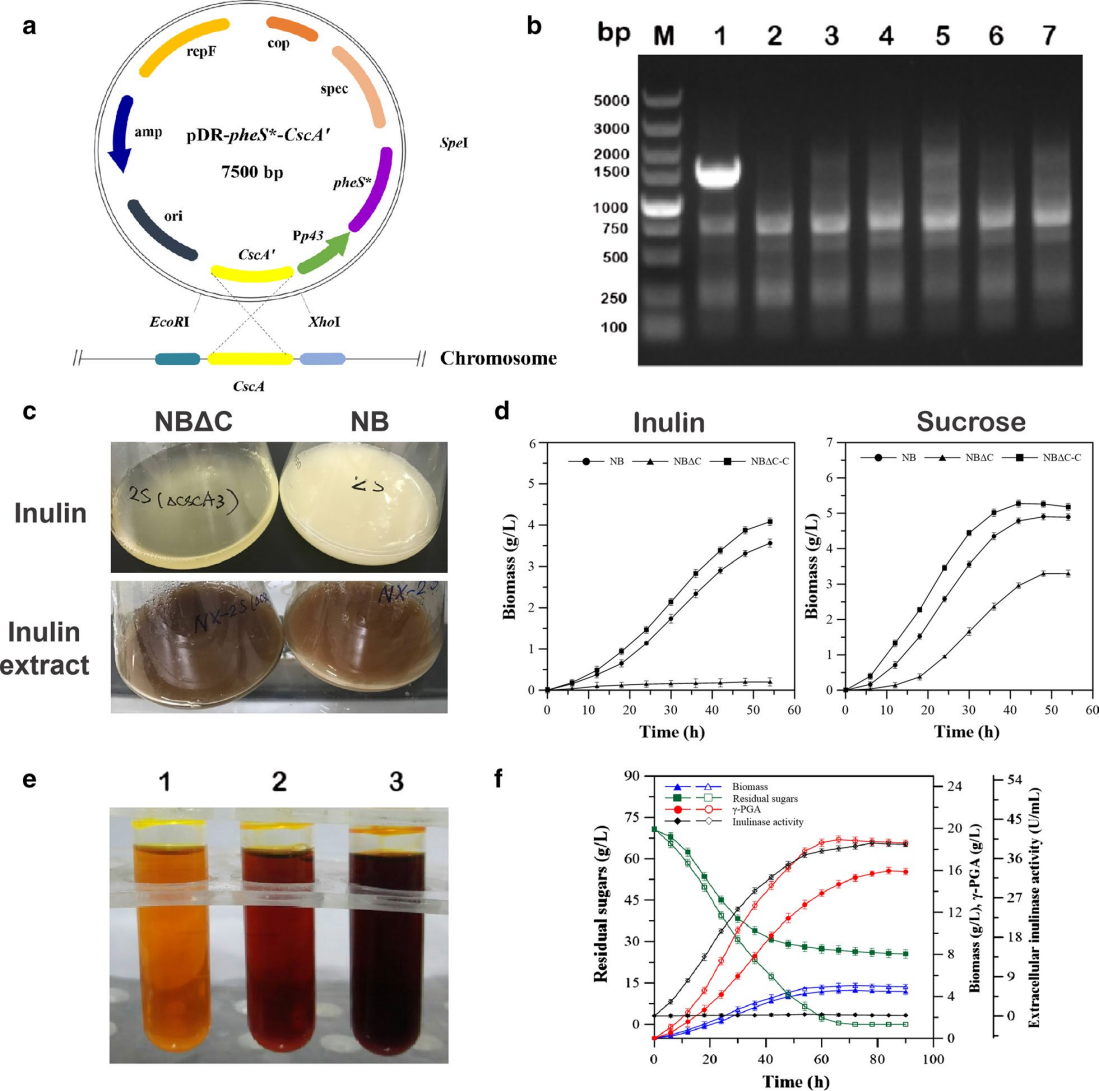
Genetic analysis of the role of the *cscA* gene in inulin metabolism of *B. amyloliquefaciens* NB. **a** Illustration of putative *cscA* disruption process. **b** PCR confirmation of the *cscA* disruption. Lane M: DL15,000 marker; lane 1: the control strain NB; lanes 2–7: *cscA* deletion mutants NBΔC. **c** Image of growth array of *cscA* deletion mutants using inulin or inulin extract as the sole carbon source. **d** Growth assay of *cscA* deletion variant NBΔC and complementation transformant NBΔC-C supplemented with inulin or sucrose. **e** Inulinase activity assay of complementation transformant NBΔC-C (lane 2) and the *cscA* gene overexpressing strain, NB-C (lane 3). **f** Time-dependent profile of γ-PGA fermentation from raw inulin extract by strain NB-C. The filled symbols represent the fermentation process of wild-type strain NB and empty symbols represent the fermentation process of strain NB-C

To verify the role of *CscA* in *B. amyloliquefaciens*, we constructed a gene disruption mutant of the *CscA* gene (Fig. 5a). The *CscA* disruption was confirmed by PCR (Fig. 5b). Then, the *B. amyloliquefaciens* mutant strain, designated NBΔC, was investigated for inulin utilization in the γ-PGA fermentation medium using inulin as the sole carbon source. As shown in Fig. 5c, the cell growth of the mutant strain NBΔC cultivated on inulin was severely impaired and no γ-PGA was detected indicating a negative effect of the *CscA* disruption on inulin utilization of the *B. amyloliquefaciens* NB. In addition, for the complementation assay, the *CscA* gene from *B. amyloliquefaciens* NB was inserted into an expression plasmid, pNX01, and was then transformed into *CscA* gene deletion strains NBΔC, generating strains NBΔC-C. Complementation of the *CscA* gene in NBΔC strains restored the normal growth phenotypes of the strain on inulin (Fig. 5d). In view of the high catalytic activity of CscA toward sucrose, we further investigated sucrose utilization profile of *CscA* deletion strains NBΔC and NBΔC-C. Due to high affinity of CscA to sucrose substrate, growth assays showed that loss of the CscA gene affected the sucrose utilization by strain *B. amyloliquefaciens* NB (Fig. 5d). This indicated that there are other enzymes which act in coordination with the CscA responsible for sucrose utilization by *B. amyloliquefaciens* [34]. In addition the gene *CscA* was the key enzyme not only for inulin metabolism but also involved in sucrose metabolism in *B. amyloliquefaciens*.

Although wild-type strain NB can spontaneously secrete hydrolase CscA for inulin hydrolysis, the extracellular inulinase activity was low, resulting in residual inulin substrate and lower γ-PGA production. This may be caused by strictly regulated expression of *CscA* gene in *B. amyloliquefaciens* strain NB. A similar phenomenon was observed for levanase in *B. subtilis*. Although the levanase gene, *sacC*, is responsible for the utilization of inulin by *B. subtilis*, levanase synthesis was repressed by glucose or fructose which led to very low extracellular inulinase activity [35]. To further improve inulin utilization by *B. amyloliquefaciens* NB, a constitutive overexpression *CscA* mutant, NB-C (pNX01-*CscA*) was constructed and its inulin utilization and γ-PGA production was investigated. The strain NB-C greatly increased extracellular inulinase activity during cultivation in the γ-PGA fermentation medium. An enzyme activity of 39.54 ± 1.2 U/mL was achieved at 78 h as shown in Fig. 5f; 70 g/L of raw inulin extract from Jerusalem artichoke tubers was consumed within 66 h of fermentation. Efficient utilization of inulin led to a significant increase in γ-PGA concentration (18.95 ± 0.31 g/L) and productivity (0.29 ± 0.012 g/L/h), which were 19.2% and 63.0% higher compared with those of wild-strain under the same condition, respectively. Meanwhile, a maximum DCW value of 4.99 ± 0.36 g/L was observed after fermentation. Based on the above results, it can be concluded that the expression of *CscA* gene involved in inulin metabolism was the limiting factor for γ-PGA production in the glutamic acid-independent strain *B. amyloliquefaciens* NB. Further metabolic modification of the inulin hydrolysis module in *B. amyloliquefaciens* could not only lead to a significant γ-PGA production, but could also lead to the development of a bacterial platform for production of more high-valued bioproducts from Jerusalem artichoke biorefinery.

## Conclusions

An inulin hydrolase, CscA, was identified and isolated from a γ-PGA-producing strain *B. amyloliquefaciens* in this study. The novel inulin hydrolase possessed relatively high activity and stability toward inulin at alkaline pH condition, compared to other inulin hydrolases, making it a suitable candidate for inulin hydrolysis during inulin biorefinery. In addition, this enzyme was found to be the key enzyme responsible for inulin metabolism in *B. amyloliquefaciens* NB. Overexpression of *CscA* gene significantly enhanced the inulin consumption, resulting in a γ-PGA concentration of 18.95 ± 0.31 g/L with a 19.2% increment and a productivity of 0.29 ± 0.012 g/L/h.

## Additional file

**Additional file 1: Figure S1.** Alignment of the amino acid sequences of CscA and other sucrose hydrolase and invertase. **Figure S2.** Product analysis of CscA with inulin and sucrose substrate. 50 g/L of inulin solution was incubated with 3 mg of purified CscA per mL at 55°C, pH 7.5 for 5 h. (a) Preparative hydrolysis of inulin with purified CscA enzyme by HPLC analysis; (b) hydrolysis of sucrose with purified CscA enzyme by HPLC analysis.

## Abbreviations

γ-PGA: poly-(γ-glutamic acid)
DCW: dry cell weight
*K*_m_: Michaelis–Menten constant
*V*_max_: maximum reaction velocity
FOSs: fructooligosaccharides
*k*_cat_: turnover number
IPTG: isopropyl-β-d-1-thiogalactopyranoside
RSM: response surface methodology
