# Supplementary material for: Systematic unravelling of the inulin hydrolase from Bacillus amyloliquefaciens for efficient conversion of inulin to poly-(γ-glutamic acid)
Source: Biotechnol Biofuels. 2019 Jun 13;12:145. doi: 10.1186/s13068-019-1485-9 (PMC6563369; doi:10.1186/s13068-019-1485-9)
Supplement: Supplementary file 1 — Additional file 1: Figure S1. Alignment of the amino acid sequences of CscA and other sucrose hydrolase and invertase. Figure S2. Product analysis of CscA with inulin and sucrose substrate. 50 g/L of inulin solution was incubated with 3 mg of purified CscA per mL at 55°C, pH 7.5 for 5 h. (a) Preparative hydrolysis of inulin with purified CscA enzyme by HPLC analysis; (b) hydrolysis of sucrose with purified CscA enzyme by HPLC analysis. [file 13068_2019_1485_MOESM1_ESM.doc]

**Additional Information**

**Systematic unravelling of the inulin hydrolase from *Bacillus amyloliquefaciens* for efficient conversion of inulin to poly-(γ-glutamic acid)**

Yibin Qiu ^1, 2^, Yifan Zhu ^1, 2^, Yijing Zhan ^3^,Yatao Zhang ^1, 2^, Yuanyuan Sha ^1, 2^, Yijing Zhan ^1, 2^, Zongqi Xu ^1, 2^, Sha Li ^1, 2^, Xiaohai Feng ^1, 2^, Hong Xu ^1, 2*^

1. State Key Laboratory of Materials-Oriented Chemical Engineering, Nanjing 211816, China

2. College of Food Science and Light Industry, Nanjing Tech University, Nanjing 211816, China

3. Nanjing Shineking Biotech Co., Ltd, Nanjing 210061, China

* Corresponding Author

Hong Xu; Nanjing Tech University; Tel/Fax: +86-25-58139433;

E-mail address: xuh@njtech.edu.cn (Hong Xu)

**Figures**

**
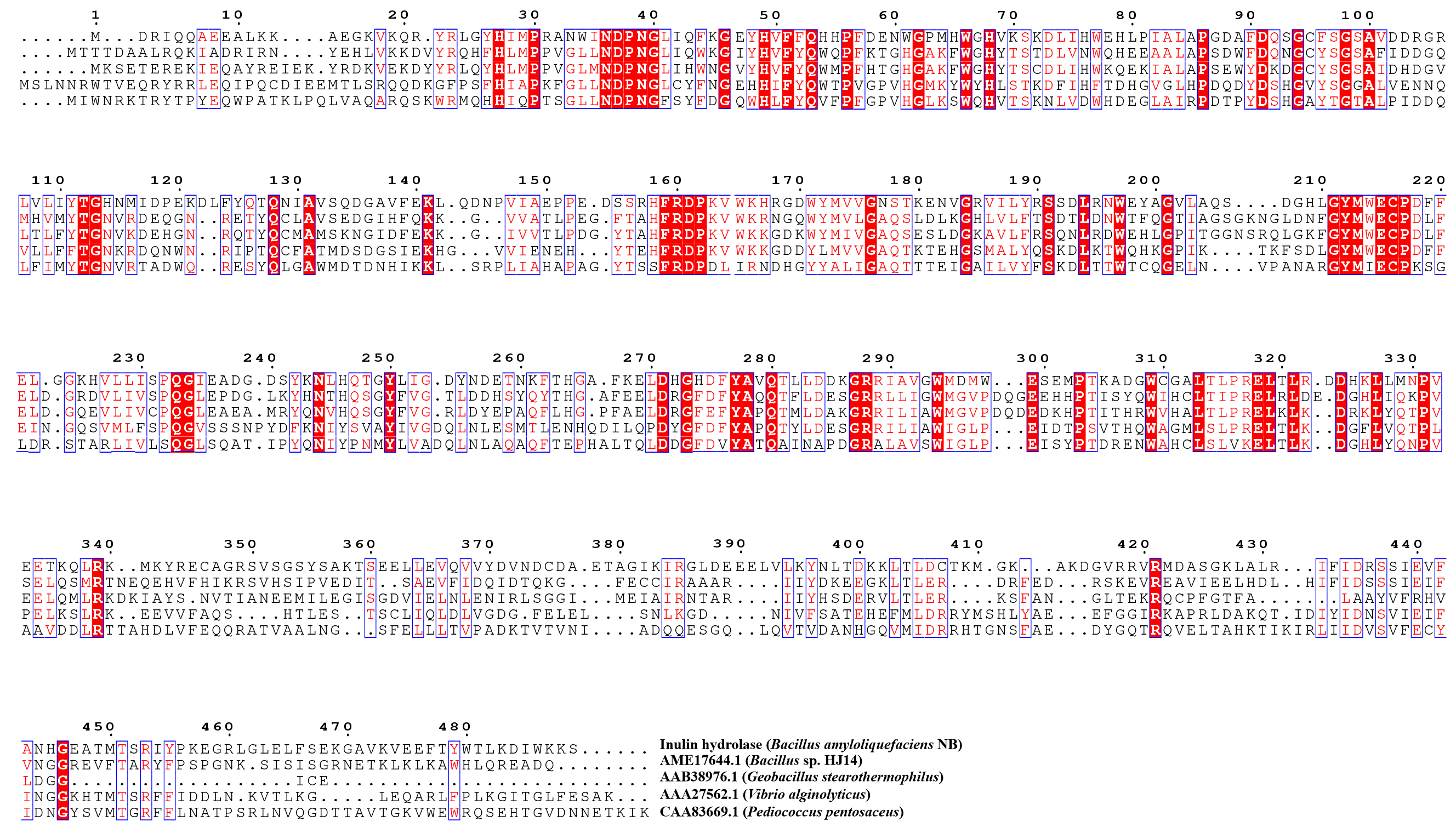
**

**Figure S1.** Alignment of the amino acid sequences of CscA and other sucrose hydrolase or invertase.

**(a) (b)**


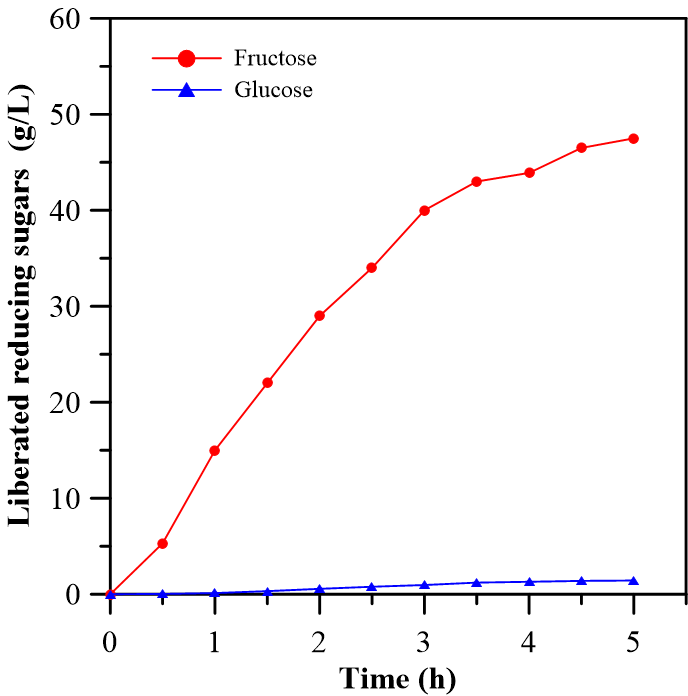

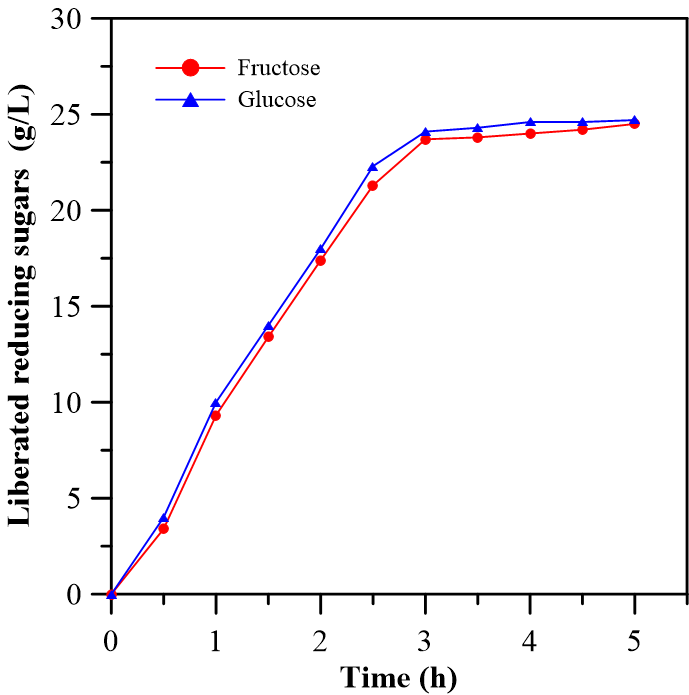


**Figure S2.** Product analysis analysis of CscA with inulin and sucrose substrate. 50 g/L of inulin solution was incubated with 3 mg of purified CscA per mL at 55℃, pH7.5 for 5 h. (a) Preparative hydrolysis of inulin with purified CscA enzyme by HPLC analysis; (b) Hydrolysis of sucrose with purified CscA enzyme by HPLC analysis
